# Flexible Magnetoelectric Device Based on VB_2_/PVDF/Metglass for Real-Time Monitoring of External Short Circuits in Lithium-Ion Batteries

**DOI:** 10.3390/polym18192314

**Published:** 2026-09-22

**Authors:** Yang Zhou, Longtai Jiang, Tao Liu, Lei Zhang, Qiang Liu, Qian Zhang

**Affiliations:** 1 School of Electronic Science and Engineering, University of Electronic Science and Technology of China, Chengdu 611731, China; yzhou2024@uestc.edu.cn; 2 School of Materials and Energy, University of Electronic Science and Technology of China, Chengdu 611731, China; 15877887034@163.com (L.J.); qiangrye@163.com (Q.L.); 3 Suzhou Coreunion Technology Co., Ltd., Suzhou 215131, China; 18182830953@163.com (T.L.); 15522643509@163.com (L.Z.); 4 School of Integrated Circuit Science and Engineering, University of Electronic Science and Technology of China, Chengdu 611731, China

**Keywords:** magnetoelectric film material, flexible device, ESC warning monitoring

## Abstract

Lithium-ion batteries are susceptible to external short-circuit (ESC) faults during charging and discharging, which induce a sudden current surge and a consequent drastic change in the surrounding magnetic field, posing a serious threat to battery safety. In this study, a VB_2_/PVDF-TrFE/Metglass magnetoelectric device was developed for real-time monitoring of ESC faults. By optimizing the VB_2_ doping ratio, the composite film with 7 wt% VB_2_ exhibited the highest β-phase content (*F_β_* = 88.03%) and optimal piezoelectric performance. After integration with Metglass, the device demonstrated stable magnetoelectric response within 0–12 kHz, with optimal performance at 11 kHz. It responds to ESC events with high sensitivity and accuracy, delivering stable signals upon each trigger and release without omission, with response times of 0.64 ms and 0.52 ms. The device is self-powered and flexible, enabling magnetic-field-to-voltage conversion without an external power supply. These results indicate its promising potential for early warning and online monitoring of external short-circuit faults in batteries.

## 1. Introduction

The growing energy demand and the large-scale integration of renewable energy have driven the rapid development of efficient, safe, and sustainable energy storage technologies. Among various energy storage systems, vanadium redox flow batteries have attracted considerable attention owing to their scalability and long cycle life, while lithium-ion batteries have become the mainstream choice for electric vehicles and large-scale energy storage systems because of their high energy density and long cycle life [1]. Lithium-ion batteries have been widely adopted in electric vehicles and large-scale energy storage systems, owing to their high energy density, long cycle life, and low self-discharge rate [2,3]. However, various safety challenges arise during charging and discharging processes, among which external short-circuit faults are particularly critical. When an ESC occurs, the loop resistance drops sharply, resulting in an instantaneous surge in discharge current and a rapid temperature rise inside the cell, which may further trigger thermal runaway and lead to fires or even explosions, posing serious threats to personal safety and equipment reliability [4,5]. Therefore, rapid and accurate real-time monitoring of ESC faults is of great significance.

Currently, battery management systems typically employ electrical parameters such as voltage, current, and temperature for fault diagnosis [6,7]. However, conventional electrical monitoring methods suffer from multiple inherent limitations when addressing ESC faults: (1) fault identification strategies based on static thresholds are prone to false alarms or missed detections under complex operating conditions, making it difficult to balance sensitivity and specificity [8,9]; (2) active sensors require external power supplies, increasing system power consumption and thermal management burden [3,6]; (3) the routing of signal transmission cables restricts sensor miniaturization and flexible integration [10]. Moreover, existing methods generally exhibit relatively long response times, which hinder the early detection of transient short-circuit surges. Therefore, there is an urgent need to develop a flexible sensing scheme with rapid response, low power consumption, and easy system integration for efficient real-time early warning of ESC events.

Magnetoelectric composite materials have demonstrated great potential in non-contact magnetic field sensing and self-powered monitoring, owing to their unique magneto-mechano-electric coupling effect [11,12,13]. Among them, poly(vinylidene fluoride-trifluoroethylene) (abbreviated as PVDF-TrFE), as a typical piezoelectric polymer, exhibits excellent flexibility, film-forming ability, and piezoelectric activity, making it an ideal matrix material for constructing flexible magnetoelectric devices [14,15]. However, the electroactive β-phase content in pristine PVDF-TrFE is relatively low, which limits its piezoelectric response sensitivity. To address this issue, researchers have attempted to induce β-phase formation by incorporating nanofillers to enhance the piezoelectric performance [16]. Commonly employed magnetostrictive materials include the rare-earth alloy Terfenol-D [17] with an ultra-large magnetostrictive coefficient, the amorphous alloy Metglass [18] combining excellent flexibility with high permeability, and ferrite materials (e.g., NiFe_2_O_4_, CoFe_2_O_4_ [19]) featuring good chemical stability and low cost. By employing various connectivity schemes, such as the 0-3 type (particle dispersion) or 2-2 type (lamellar composite), the piezoelectric phase and magnetostrictive phase can be integrated to construct magnetoelectric coupling structures that enable sensitive responses to weak magnetic field variations [13,20].

Based on the above considerations, this study designs a 2-2 type laminated flexible self-powered magnetoelectric device based on vanadium diboride (VB_2_)/PVDF-TrFE/Metglass for real-time monitoring of external short-circuit (ESC) faults in lithium-ion batteries. VB_2_, featuring polar functional groups including hydroxyl (–OH), carbonyl (C=O), and amino (–NH_2_) moieties, establishes robust hydrogen-bonding interactions with PVDF-TrFE chains, thereby effectively stabilizing the electroactive β-phase and substantially enhancing the piezoelectric performance [21]. Its all-organic nature further ensures favorable interfacial compatibility and straightforward processability within the composite system. Metglass, possessing a high magnetostrictive coefficient and excellent flexibility, serves dually as a magnetic-field-sensitive layer for efficient magneto-mechano-electric transduction and as a conductive bottom electrode, while forming an adhesive-free, high-integrity interface with PVDF-TrFE that facilitates effective mechanical stress transfer. The influence of VB_2_ doping ratios (3–11 wt%) on the crystallization behavior and piezoelectric response of PVDF-TrFE was systematically investigated, and the optimal composition was determined to be 7 wt%, at which the composite film achieved the highest β-phase content (*F_β_* = 29.24%) and optimal overall piezoelectric performance. The optimized piezoelectric composite film was subsequently laminated with the flexible Metglass magnetostrictive substrate to construct a magnetoelectric composite structure and was encapsulated into a flexible sensing device. Experimental results demonstrate that the device exhibits stable magnetoelectric response across a broad frequency range of 0–12 kHz, with a peak output at 11 kHz, and achieves millisecond-level response times (0.64 ms and 0.52 ms) with excellent reliability in ESC simulation tests, highlighting its considerable potential as a novel flexible sensing solution for battery safety management.

## 2. Materials and Methods

### 2.1. Materials

PVDF-TrFE powder with a VDF/TrFE molar ratio of 80/20 (Mw ≈ 520,000) was supplied by Akema Piezotech (Paris, France). VB_2_ powder was purchased from Shanghai Macklin Biochemical Co., Ltd. (Shanghai, China). The butanone was purchased from Chengdu Cologne Chemical Co., Ltd. (Chengdu, China). ITO (indium tin oxide) conductive glass was purchased from Luoyang Guluo Glass Co., Ltd. (Luoyang, China). The Metglass (Fe-based nanocrystalline alloy, 1K107B) was purchased from China Amorphous Technology Co., Ltd. (Foshan, China).

### 2.2. Fabrication of VB_2_/PVDF-TrFE Composites

PVDF-TrFE powder was completely dissolved in butanone via magnetic stirring at room temperature, and VB_2_ powder was then dispersed in the resulting solution at varying mass ratios (97:3, 95:5, 93:7, 91:9, and 89:11 wt%) to yield homogeneous suspensions. Each formulation was subsequently deposited onto either ITO-coated glass or flexible Metglass substrates via a slot-die coating process (coating speed: 55 mm·s^−1^; doctor blade height: 220 μm), followed by vacuum drying (Shanghai Yiheng Scientific Instrument Co., Ltd., Shanghai, China) at 10 Pa for 8 min to obtain dried films with a nominal thickness of 20 μm. The VB_2_/PVDF-TrFE composite films deposited on ITO substrates were designated for comprehensive characterization to evaluate their physicochemical properties, whereas their counterparts on Metglass substrates were employed for the fabrication, encapsulation, and performance assessment of magnetoelectric devices. All film samples were subsequently annealed at 140 °C for 40 min and then subjected to in situ electrical poling (gate voltage: 7500 V; source voltage: 3500 V; poling time: 15 min; poling temperature: room temperature) to induce piezoelectric activity. A silver (Ag) top electrode (Length × Width = 2 cm × 1 cm) was applied onto each VB_2_/PVDF-TrFE film surface via screen printing, with the underlying ITO-coated glass or flexible Metglass substrate serving as the bottom electrode. The fabricated current sensors are shown in Figure 1, which presents schematic diagrams of the VB_2_/PVDF-TrFE composite films prepared with varying VB_2_ mass ratios, along with photographic images of the films deposited on different substrates. For the VB_2_/PVDF-TrFE/Metglass magnetoelectric devices, the active sensing area is 2 cm × 1 cm; the final layer thickness of the VB_2_/PVDF-TrFE composite film is approximately 24 μm; and the encapsulation material is polyimide (PI) tape.

### 2.3. Characterization

Scanning electron microscopy (SEM) and energy-dispersive spectroscopy (EDS) analyses were performed using a ZEISS GeminiSEM 300 system (Carl Zeiss, Oberkochen, Germany) (magnification: 5 K–200 K). Crystallization behavior was characterized by X-ray diffraction (XRD) using a Rigaku Ultima IV diffractometer (Rigaku, Tokyo, Japan) (scanning angle: 5–90°; scanning speed: 2°/min). Thermal analysis curves were measured with a Netzsch DSC 200 F3 (Netzsch, Selb, Germany) differential scanning calorimeter (Germany) at a heating/cooling rate of 10 °C/min. Fourier transform infrared (FTIR) spectra were collected using a Thermo Scientific Nicolet iN 10 infrared microscope (Thermo Fisher Scientific, Waltham, MA, USA) (wavenumber range: 400–4000 cm^−1^). Piezoelectric signals were recorded with an NI 9234 voltage acquisition card (National Instruments, Austin, TX, USA). Magnetoelectric signals were jointly acquired using a DG4162 function generator (RIGOL Technologies, Inc., Beijing, China), an ATA-304 power amplifier (Xi’an Aigtek Electronic Technology Co., Ltd., Xi’an, China), an HLY30-50 Helmholtz coil (Changchun Yingpu Magnetoelectric Technology Development Co., Ltd., Changchun, China), and an NI 9234 data acquisition card. The charge/discharge cycling of the lithium-ion battery was driven by a 5V2A8C LANHE battery test system (Wuhan LAND Electronic Co., Ltd., Wuhan, China).

## 3. Results and Discussion

### 3.1. Design Concept and Work Principle

As illustrated in Figure 2, VB_2_ was doped into PVDF-TrFE to enhance its piezoelectric properties, and the resulting VB_2_/PVDF-TrFE composite film was coated onto a Metglass substrate (Length × Width = 2 cm × 1 cm) to construct a magnetoelectric signal transduction device. The operating principle of this device is based on the magnetostrictive effect of Metglass: when Metglass undergoes strain, the strain is transferred to the VB_2_/PVDF-TrFE piezoelectric layer, thereby generating an electrical signal output. Based on this mechanism, the sensor was employed for real-time monitoring of external short-circuit faults in batteries. When an external short circuit occurs, the sharp increase in fault current induces a drastic change in the magnetic field around the battery pack and the VB_2_/PVDF-TrFE/Metglass composite sensor, driving the Metglass layer to produce magnetostrictive deformation. This deformation is then transferred to the piezoelectric layer, inducing accumulated charges on the electrode surface and generating a piezoelectric voltage, which is simultaneously recorded and monitored by an external acquisition system.

### 3.2. Characterization of the VB_2_/PVDF-TrFE Composite Films

Figure 3 presents the SEM-EDS characterization results of the 7 wt% VB_2_/PVDF-TrFE composite film. The cross-sectional morphology and elemental distribution of the VB_2_/PVDF-TrFE composite film were characterized by SEM-EDS. As shown in Figure 3a,b, the cross-sectional SEM image reveals a dense and uniform film structure, free of visible cracks, voids, or delamination defects, indicating good consistency in the coating and film-forming process. As shown in Figure 3c–h, EDS elemental mapping results show that C and F, as characteristic elements of the PVDF-TrFE matrix, exhibit continuous and homogeneous distribution across the entire scanned region, confirming the formation of an intact continuous polymer phase. N, serving as the characteristic marker for the VB_2_ filler, displays a relatively dispersed distribution within the matrix, although slight localized enrichment is observable, which may be attributed to agglomeration induced by van der Waals forces among nanoparticles. No significant elemental segregation or abrupt concentration gradients are observed at the VB_2_/PVDF-TrFE phase interfaces, suggesting good interfacial wettability and compatibility between VB_2_ and PVDF-TrFE, without notable phase separation or structural discontinuity. This uniform filler dispersion and robust interfacial bonding provide a reliable structural foundation for efficient stress transfer and charge accumulation in the composite film under external magnetic or mechanical stimuli, thereby ensuring its synergistic response performance in magnetoelectric sensing applications.

Figure 4 shows the XRD patterns of VB_2_/PVDF-TrFE composite films with different VB_2_ doping contents. X-ray diffraction (XRD) was performed to investigate the effect of VB_2_ doping concentration on the crystallization behavior of VB_2_/PVDF-TrFE composite films. The diffraction peaks at 18.3° and 26.7° are assigned to the (020) and (021) planes of the α-phase, respectively [22]. With increasing VB_2_ content, the α-phase peaks at 26.7° and 18.3° progressively diminish, indicating a decrease in the α-phase fraction. Conversely, the β-phase diffraction peaks at 20.3° and 35.8°, corresponding to the (110)/(200) and (001) planes [23], increase markedly with VB_2_ loading from 3 to 11 wt%, while the peak at 41.2° remains essentially unchanged. These results suggest that VB_2_ promotes the α-to-β conformational transition, with β-phase crystallinity improving with higher VB_2_ content. This transition is primarily attributed to interfacial interactions between VB_2_ nanoparticles and PVDF chains, wherein the surface polarity of the nanofillers induces preferential all-trans (TTTT) chain conformation during crystallization, enriching the β-phase. The enhanced electroactive β-phase content directly underpins the improved piezoelectric response of the composite films.

The XRD diffraction peak intensity depends not only on the β-phase content but also on the overall degree of crystallinity, crystallite size, crystalline perfection, and preferred orientation. Therefore, the XRD results alone are insufficient to accurately determine the relative β-phase content. FTIR analysis was further performed to calculate the β-phase fraction so as to more accurately evaluate the effect of VB_2_ doping on β-phase formation in PVDF-TrFE.

Figure 5 presents the Fourier-transform infrared (FTIR) spectra of VB_2_/PVDF-TrFE composite films with varying VB_2_ doping contents. The characteristic absorption bands of the α-phase are located at 614, 764, 976, and 1234 cm^−1^, whereas those of the β-phase are observed at 840 and 1175 cm^−1^ [23]. As shown in Figure 5, with increasing VB_2_ content, the absorption peak intensities at 840 and 1175 cm^−1^ exhibit a non-monotonic trend of initially increasing and subsequently decreasing, indicating that the β-phase fraction first rises and then falls with higher VB_2_ doping levels. This observation suggests that an appropriate amount of VB_2_ doping effectively promotes the conformational transition of PVDF molecular chains from the non-polar α-phase to the polar β-phase. However, excessive VB_2_ loading may induce nanoparticle agglomeration, which weakens the interfacial interactions between the nanofillers and the PVDF matrix, thereby suppressing further β-phase formation.

The fraction of the polar β-phase (*F_β_*) in the VB_2_/PVDF-TrFE composite films was quantitatively determined using the Beer–Lambert law [24]:

(1) $$F_{\beta }=\frac{A_{\beta }}{\frac{K_{\beta }}{K_{\alpha }}A_{\alpha }+A_{\beta }}=\frac{A_{\beta }}{1.26A_{\alpha }+A_{\beta }}$$

where *A_β_* and *A_α_* denote the absorbance intensities of the characteristic bands at 840 cm^−1^ and 764 cm^−1^, respectively, and *K_β_* and *K_α_* are the corresponding absorption coefficients, with established values of 7.7 × 10^4^ and 6.1 × 10^4^, respectively.

The polar β-phase content (*F_β_*) of the composite films was quantitatively determined using the Beer–Lambert law, and the calculated *F_β_* values for pure PVDF-TrFE and the VB_2_/PVDF-TrFE composite films with 3 wt%, 5 wt%, 7 wt%, 9 wt%, and 11 wt% VB_2_ loading are summarized in Table 1, corresponding to 80.96%, 83.34%, 85.89%, 88.03%, 78.91%, and 75.32%, respectively. As the VB_2_ content increases from 3 wt% to 7 wt%, the absorption peak intensity at 840 cm^−1^ progressively increases, accompanied by a corresponding rise in *F_β_*, indicating that an appropriate amount of VB_2_ doping effectively promotes the conformational transition of PVDF molecular chains from the non-polar α-phase to the polar β-phase. However, further increasing the VB_2_ loading to 9 wt% and 11 wt% leads to a decline in *F_β_* to 28.99% and 28.76%, respectively, exhibiting a non-monotonic trend of initial increase followed by a decrease. This behavior is ascribed to the agglomeration of excessive VB_2_ nanoparticles within the polymer matrix, which reduces the effective interfacial contact area between the filler and the matrix, thereby weakening the nucleation-inducing effect of VB_2_ surfaces on PVDF chain crystallization and partially suppressing the formation of the β-phase. These results demonstrate that the VB_2_ doping concentration exerts a dual regulatory effect on the β-phase content of the composite films, and only within an appropriate doping window can the electroactive β-phase be effectively promoted.

Figure 6 presents the differential scanning calorimetry (DSC) curves of VB_2_/PVDF-TrFE composite films with varying VB_2_ doping contents, where Figure 6a shows the melting behavior and Figure 6b displays the crystallization behavior. In Figure 6a, Tc denotes the Curie temperature, above which the material undergoes a ferroelectric-to-paraelectric phase transition accompanied by the disordering of internal dipoles, resulting in the loss of piezoelectric activity; Tm represents the melting temperature, beyond which the polymer transitions into a viscous flow state. When the VB_2_ mass fraction ranges from 3 wt% to 11 wt%, the melting temperatures (Tm) of the composite films are consistently higher than those of pure PVDF-TrFE. Notably, this observation is consistent with the increased intensities of the β-phase diffraction peaks at 20.3° and 35.8° observed in the XRD patterns with increasing VB_2_ content, further confirming that VB_2_ acts as an effective nucleating agent during the crystallization process, promoting the crystallization of PVDF-TrFE.

Figure 6b presents the crystallization behavior of the samples with varying VB_2_ doping levels upon cooling. All six samples exhibit two distinct exothermic peaks at approximately 125 °C and 86 °C, corresponding to the crystallization temperature (Tc) and the paraelectric-to-ferroelectric phase-transition temperature, respectively. Notably, the crystallization temperatures of the VB_2_/PVDF-TrFE composite films are consistently higher than those of pure PVDF-TrFE, which can be attributed to the fact that crystalline regions generally exhibit higher transition temperatures than amorphous regions, and the heterogeneous nucleation effect of VB_2_ nanoparticles enhances the crystalline ordering of the polymer chains. However, when the VB_2_ doping content is further increased to 9 wt% and 11 wt%, the enhancement of the crystallization temperature tends to saturate or even slightly decrease. This variation is consistent with the declining trend of *F_β_* values at 9 wt% and 11 wt% observed in the FTIR analysis, further corroborating that excessive VB_2_ doping induces nanoparticle agglomeration, which weakens its nucleation-inducing effect. Collectively, the combined results from FTIR, XRD, SEM-EDS, and DSC analyses provide compelling evidence that an appropriate amount of VB_2_ doping effectively promotes the formation of the β-phase in PVDF-TrFE, whereas excessive doping suppresses further optimization of the crystallization behavior due to agglomeration effects.

### 3.3. Piezoelectric Properties of the VB_2_/PVDF-TrFE Composite Films

An electrical characterization platform was established to systematically evaluate the electrical performance of the VB_2_/PVDF-TrFE devices and to investigate the influence of various factors on their output response. A fixed compressive force of approximately 2 N was applied to the surface of the VB_2_/PVDF-TrFE device (5 cm × 5 cm VB_2_/PVDF-TrFE composite film deposited on an ITO substrate). Upon sensing the external pressure, the device generated an output voltage signal, which was captured by a voltage acquisition card connected via leads and subsequently recorded and analyzed using a computer equipped with DAQ-Express software (Version 5.1).

Figure 7 presents the output voltage signals of the VB_2_/PVDF-TrFE composite films with varying VB_2_ doping levels. The output voltage increases progressively with increasing VB_2_ content, reaching a maximum at 7 wt% VB_2_ loading. This trend is consistent with the FTIR characterization results, in which the 7 wt% VB_2_/PVDF-TrFE sample exhibited the highest *F_β_* value, indicating that the enhanced β-phase content directly contributes to the improved piezoelectric response of the composite films. However, when the VB_2_ doping level is further increased to 9 wt% and 11 wt%, the output voltage exhibits a declining trend. This phenomenon can be rationalized from two aspects. On one hand, excessive VB_2_ nanoparticles tend to agglomerate within the polymer matrix, reducing the effective interfacial contact area between the filler and the polymer, thereby weakening the interfacial polarization effect. On the other hand, the steric hindrance introduced by excessive doping impedes the ordered arrangement of PVDF molecular segments and the formation of β-phase crystals, leading to a reduction in the electroactive β-phase content within the composite films, which in turn diminishes the polarization efficiency and output performance of the piezoelectric charge.

Based on the above output voltage signal analysis, the 7 wt% VB_2_/PVDF-TrFE composite film device demonstrates the best electrical performance among all samples. Hence, it is selected as the optimal composition for the subsequent integration with Metglass to construct magnetoelectric composite materials.

### 3.4. Magnetoelectric Performance of VB_2_/PVDF-TrFE/Metglass Magnetoelectric Devices

To evaluate the magnetoelectric performance of the VB_2_/PVDF-TrFE/Metglass magnetoelectric devices, a magnetoelectric testing platform was established in this study for systematic characterization and for investigating the influence of various factors on their response behavior. The testing platform mainly consists of a function generator, a power amplifier, a Helmholtz coil, a voltage acquisition card, and a PC. Figure 8a illustrates its working principle: the coil is driven by a function generator and a power amplifier to generate an alternating magnetic field (*H_ac_*) of 1590 A·m^−1^ (≈20 Oe) along the length direction of the VB_2_/PVDF-TrFE/Metglass magnetoelectric devices. The magnetoelectric composite sensor is positioned at the center of the Helmholtz coil, and its top and bottom electrodes were connected to the positive and negative terminals of Channel 1 of the voltage acquisition card. Upon exposure to the external alternating magnetic field, a corresponding voltage output signal is generated across its top and bottom surfaces. This signal is captured by the voltage acquisition card via leads and subsequently recorded and analyzed using a computer equipped with DAQ-Express software.

In practical scenarios, electromagnetic interference or stray magnetic fields originating from electrical equipment (such as power transmission lines and household appliances) often span a wide frequency spectrum [25], and the magnetoelectric response measured at a single frequency is insufficient to fully reflect the sensing performance of the device under real operating conditions. Therefore, it is necessary to systematically investigate the magnetoelectric response characteristics of the VB_2_/PVDF-TrFE/Metglass magnetoelectric devices across a broad frequency range. To this end, the output voltage frequency of the function generator was adjusted to generate alternating magnetic fields of varying frequencies while maintaining a constant magnetic field amplitude, enabling a comprehensive exploration of the magnetoelectric response behavior of the device over a wide frequency range.

As shown in Figure 8b, the magnetoelectric performance of the VB_2_/PVDF-TrFE/Metglass magnetoelectric device increases with increasing frequency under low-frequency magnetic fields. As presented in Figure 8c, within the high-frequency range of 1–11 kHz, the magnetoelectric performance of the device improves significantly with increasing frequency. However, when the frequency reaches 12 kHz, the magnetoelectric performance declines. At 13 kHz, the device fails to operate properly, which is attributed to the combined effects of the power amplifier bandwidth and the inductive impedance of the Helmholtz coil, which prevent the effective generation of a stable voltage signal at this frequency. Consequently, the VB_2_/PVDF-TrFE/Metglass magnetoelectric device operates properly within the high-frequency range of 0–12 kHz, with the optimal magnetoelectric performance achieved at 11 kHz.

This operating frequency range covers the typical electromagnetic interference bands generated by power transmission lines (50/60 Hz power frequency and its harmonics), switching power supplies (tens to hundreds of kilohertz), and household appliances (such as variable-frequency motors and wireless charging devices), indicating that the VB_2_/PVDF-TrFE/Metglass magnetoelectric device exhibits stable magnetoelectric response capability under the aforementioned practical scenarios.

### 3.5. Applications of VB_2_/PVDF-TrFE/Metglass Magnetoelectric Devices in Real-Time Monitoring of External Short Circuits (ESC) in Batteries

During the charging and discharging processes of lithium-ion batteries, the current typically exhibits instantaneous fluctuations. Accordingly, the VB_2_/PVDF-TrFE/Metglass magnetoelectric device can be employed to detect current variations in real time, enabling rapid response to external short-circuit (ESC) faults in battery systems. Battery faults are generally characterized by abnormal deviations in input/output current and voltage. When an ESC occurs, the current surges abruptly, inducing a significant variation in the magnetic field surrounding the cell, which can be effectively captured by the VB_2_/PVDF-TrFE/Metglass magnetoelectric device. As shown in Figure 9a, the device is fixed above a pouch cell for ESC monitoring, while the battery current and voltage are simultaneously recorded by a LANHE battery test system. During the charging/discharging process, ESC faults are simulated by manually short-circuiting the positive and negative terminals of the battery.

As presented in Figure 9b, ten ESC simulation experiments were conducted by manually connecting and disconnecting the positive and negative terminals of the battery under discharge conditions to induce ESC events. During each simulation, the battery current underwent drastic fluctuations, and the VB_2_/PVDF-TrFE/Metglass magnetoelectric device correspondingly generated two voltage signals: the first signal corresponds to terminal connection (ESC trigger), and the second corresponds to terminal disconnection (ESC release), with the time interval between the two signals representing the ESC duration. In the figure, the blue numbers “1, 3, 5,…, 19” denote the output voltage peaks of the device at the moments of ESC triggering, while the red numbers “2, 4, 6,…, 20” denote those at the moments of ESC release. These results demonstrate that the VB_2_/PVDF-TrFE/Metglass magnetoelectric device provides a stable and reproducible voltage response to every ESC event, with signal outputs precisely corresponding to each instance of ESC onset and release, without any missed detection or false response. This verifies the reliability and real-time responsiveness of the device for external short-circuit fault monitoring in battery systems.

Figure 9c,d present the locally enlarged time regions corresponding to Signal 1 and Signal 2, respectively, to further analyze the response time characteristics of the device to ESC events. By extracting the time coordinates of the output voltage peaks, the signal delay at the moments of ESC onset and release can be determined, enabling quantitative evaluation of the response speed and real-time monitoring capability of the device. As indicated by the “Start” and “Warning” markers shown in Figure 9c,d, when an ESC event occurs in the pouch cell, the VB_2_/PVDF-TrFE/Metglass magnetoelectric device generates voltage signals with response times of 0.64 ms and 0.52 ms, respectively. These results demonstrate that the device possesses a millisecond-level response time, enabling rapid detection of external short-circuit faults in batteries and satisfying the timing requirements for early fault warning in practical engineering applications. Furthermore, the device enables passive magnetic-field-to-voltage conversion without the need for an external power supply, offering the distinct advantage of self-powered sensing, which effectively reduces system power consumption and wiring complexity. These features endow the magnetoelectric device with considerable practical value and promising application prospects in scenarios demanding high safety and real-time performance, including electric vehicle battery management systems, energy storage power stations, and portable electronic devices.

## 4. Conclusions

In this study, VB_2_/PVDF-TrFE composite films with varying VB_2_ doping levels (3–11 wt%) were successfully fabricated and systematically characterized. Through structural, thermal, and electrical performance analyses, the regulatory mechanism of VB_2_ on the crystallization behavior and piezoelectric response of the material was clarified, and its potential for magnetoelectric sensing and battery fault monitoring was verified. XRD and FTIR analyses demonstrated that the incorporation of VB_2_ significantly promotes the formation of the polar β-phase in PVDF-TrFE. At a VB_2_ content of 7 wt%, the β-phase content (*F_β_*) reached a maximum of 88.03%, corresponding to optimal piezoelectric performance. DSC analysis further confirmed that VB_2_ nanoparticles act as heterogeneous nucleating agents during crystallization, enhancing the crystallinity and thermal stability of the composite films.

On this basis, the 7 wt% VB_2_/PVDF-TrFE composite film was integrated with a Metglass substrate to construct a flexible magnetoelectric composite device. Magnetoelectric performance tests revealed that the device exhibits a stable response within 0–12 kHz, with optimal performance at 11 kHz. In battery external short-circuit (ESC) simulation experiments, the device responded to each ESC event with high sensitivity and accuracy, achieving millisecond-level response times of 0.64 ms and 0.52 ms without omission. Moreover, the device is self-powered and flexible, enabling magnetic-field-to-voltage conversion without an external power supply. These results demonstrate that the VB_2_/PVDF-TrFE/Metglass magnetoelectric device holds great promise for early warning monitoring of external short-circuit faults in batteries, offering a novel sensing solution for the safety management of electric vehicles, energy storage systems, and portable electronic devices.

## Figures and Tables

**Figure 1:**
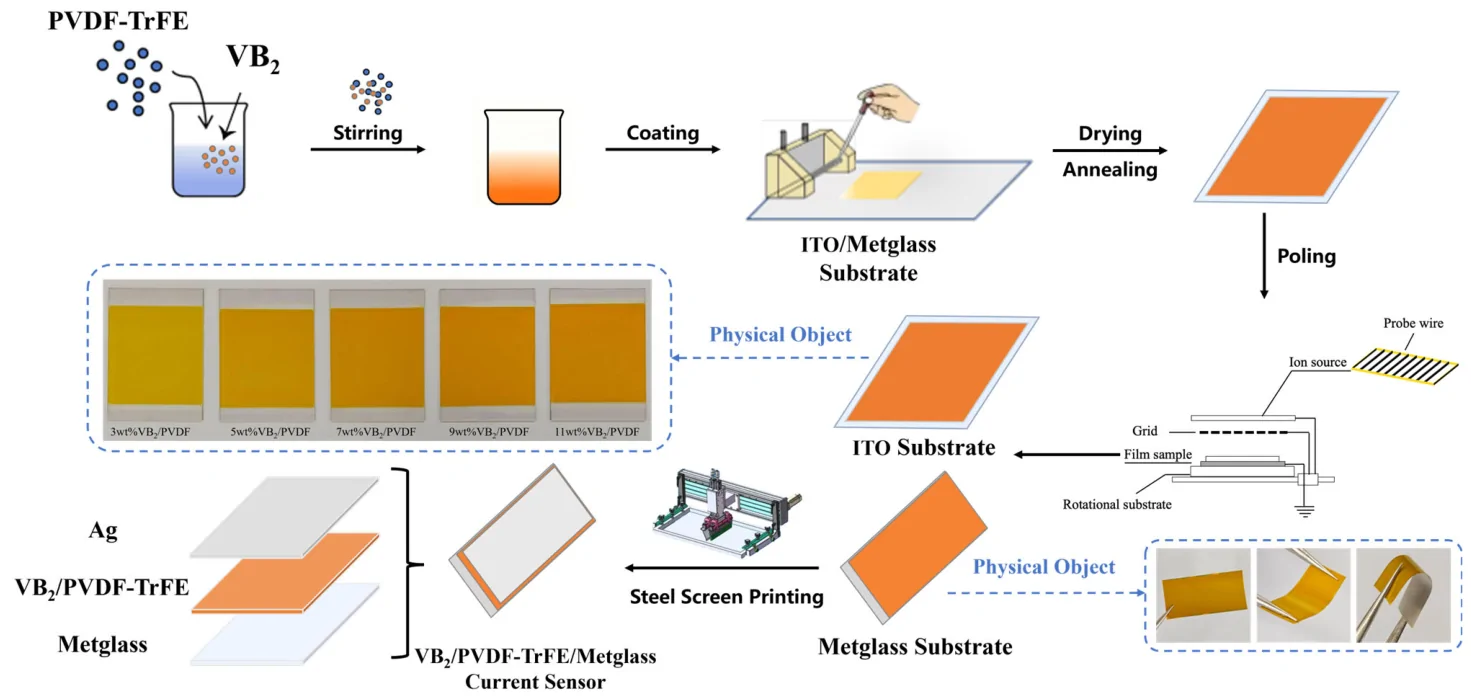
Fabrication process of VB_2_/PVDF-TrFE composite and sensors.

**Figure 2:**
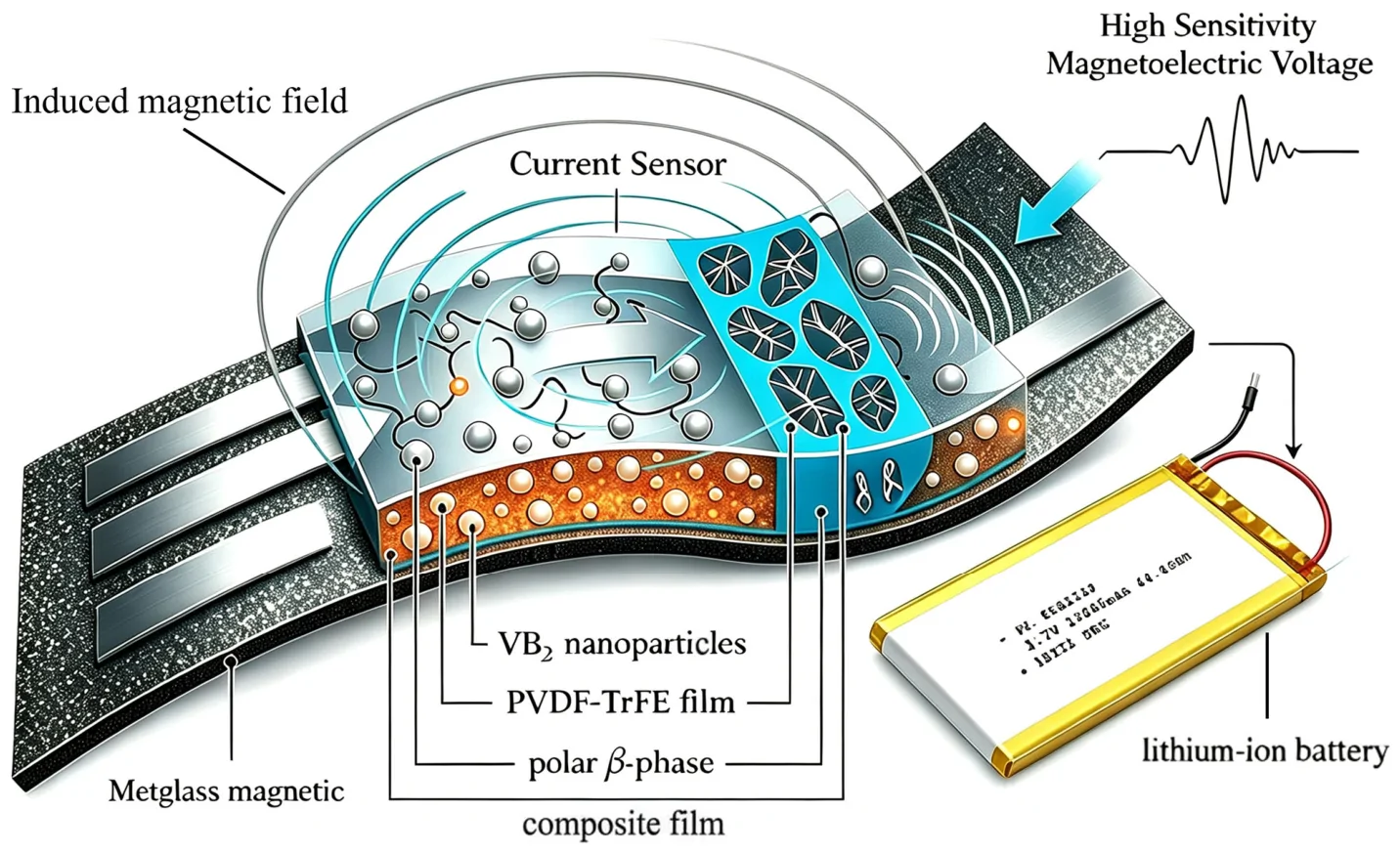
Conceptual diagram of the VB_2_/PVDF-TrFE/Metglass composite sensor.

**Figure 3:**
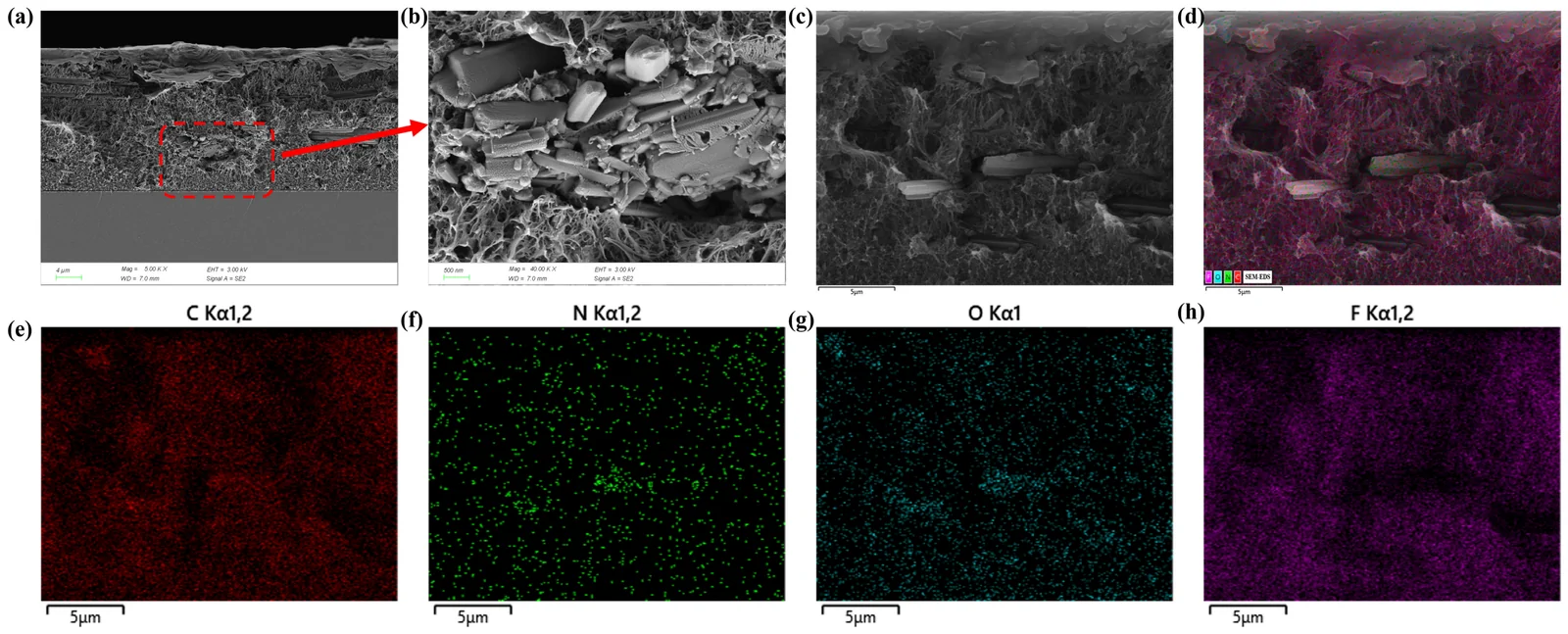
Microstructure and elemental distribution of the 7 wt% VB_2_/PVDF-TrFE composite film: (**a**) the 5k× SEM image showing the overall surface morphology and dispersion of VB_2_ particles; (**b**) the 40k× SEM image showing the interfacial bonding between VB_2_ particles and the PVDF-TrFE matrix as well as the particle size; (**c**) EDS cross-sectional scanning position indicating the mapped area; (**d**) overall EDS elemental mapping of the cross section; (**e**–**h**) EDS elemental maps of C, N, O, and F, respectively, showing the spatial distribution of each element in the composite film.

**Figure 4:**
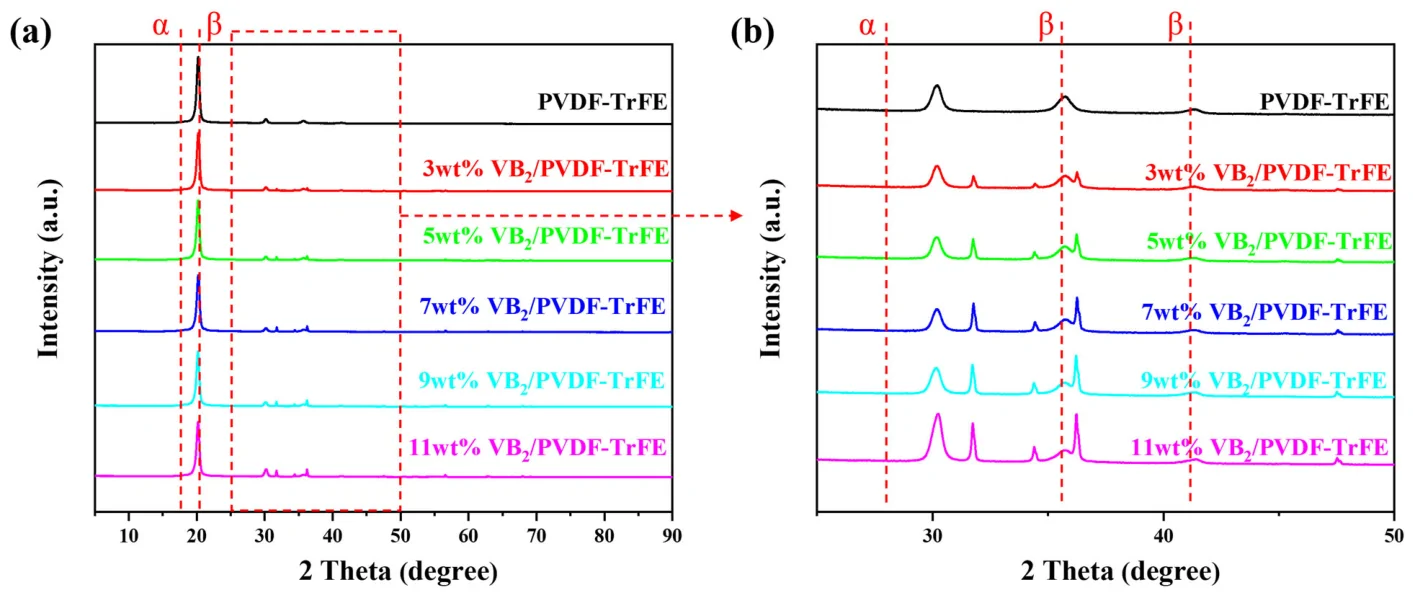
XRD patterns of VB_2_/PVDF-TrFE composite films: (**a**) full pattern in the 2θ range of 10–90°; (**b**) magnified view in the 2θ range of 25–50°.

**Figure 5:**
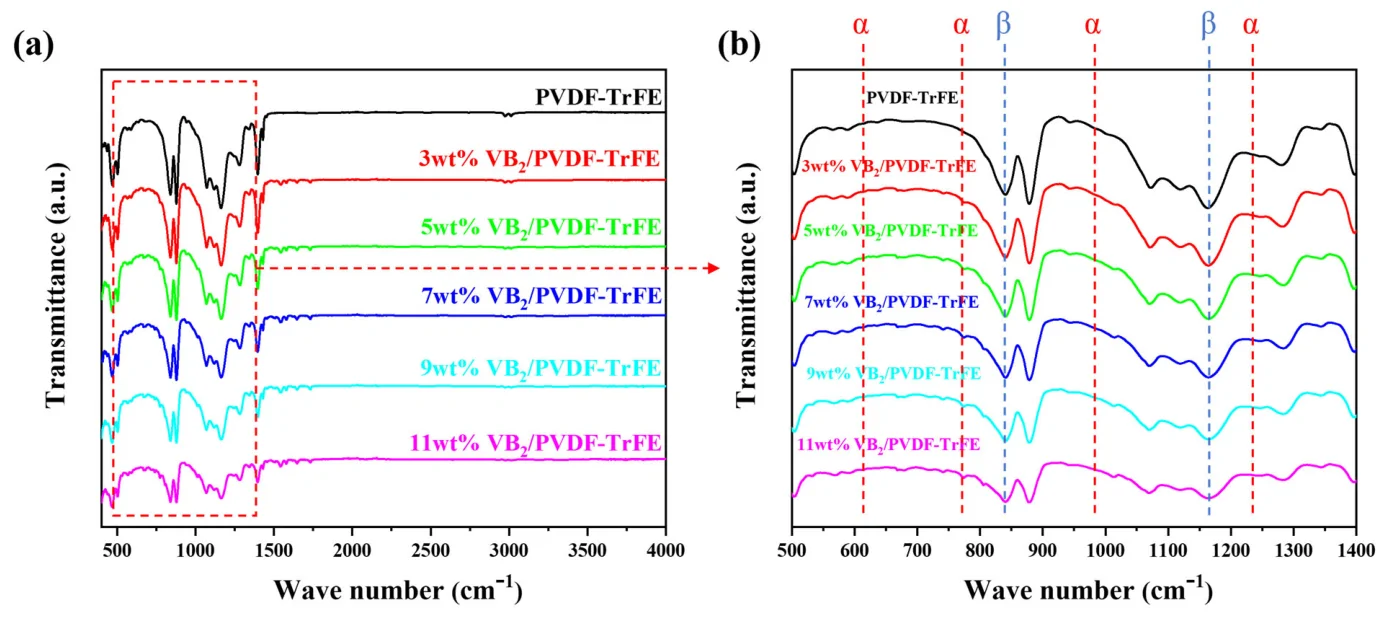
FTIR patterns of VB_2_/PVDF-TrFE film. (**a**) Wavenumber range of 200–4000 cm^−1^; (**b**) magnified view in the wavenumber range of 500–1400 cm^−1^.

**Figure 6:**
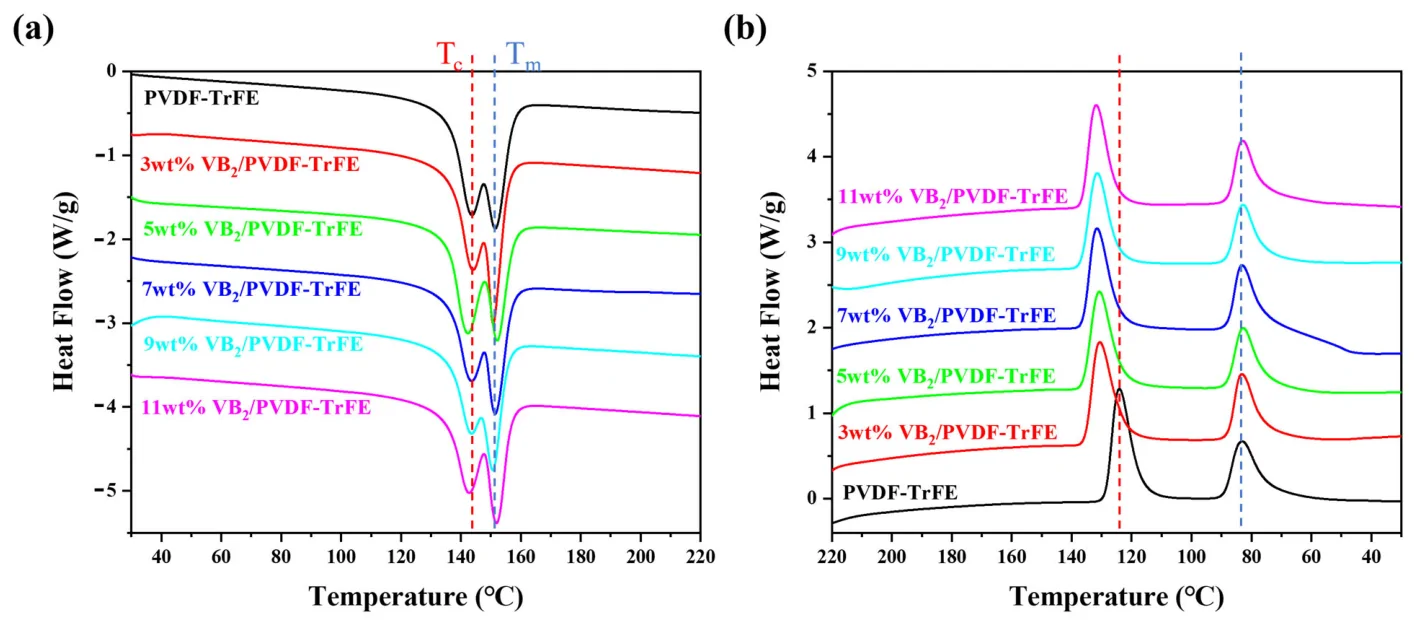
(**a**) Melting and (**b**) crystallization behaviors of the VB_2_/PVDF-TrFE composite films.

**Figure 7:**
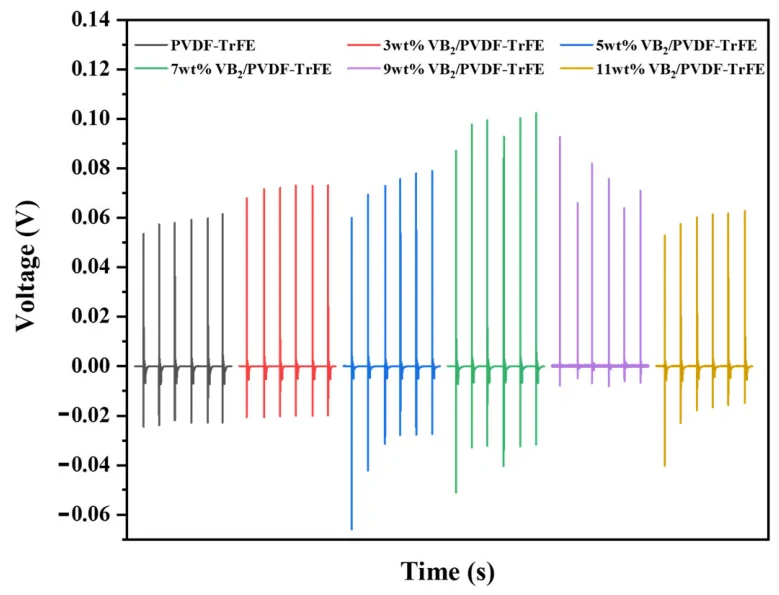
Output electrical signals of VB_2_/PVDF-TrFE composite films with different VB_2_ doping levels.

**Figure 8:**
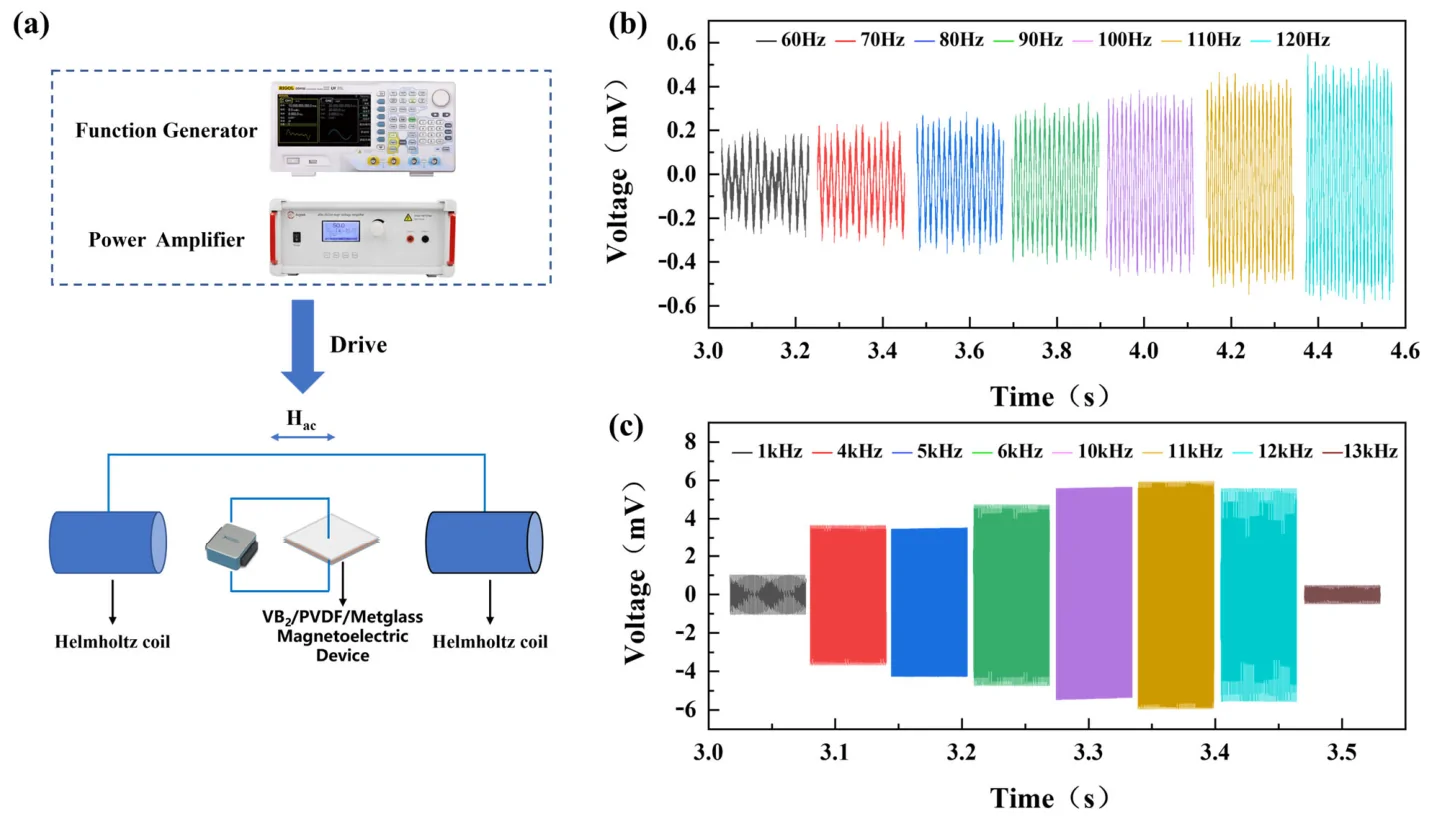
(**a**) Schematic illustration of the magnetoelectric testing platform; (**b**) magnetoelectric response of the VB_2_/PVDF-TrFE/Metglass magnetoelectric device under low-frequency magnetic fields; (**c**) magnetoelectric response of the VB_2_/PVDF-TrFE/Metglass magnetoelectric device under high-frequency magnetic fields.

**Figure 9:**
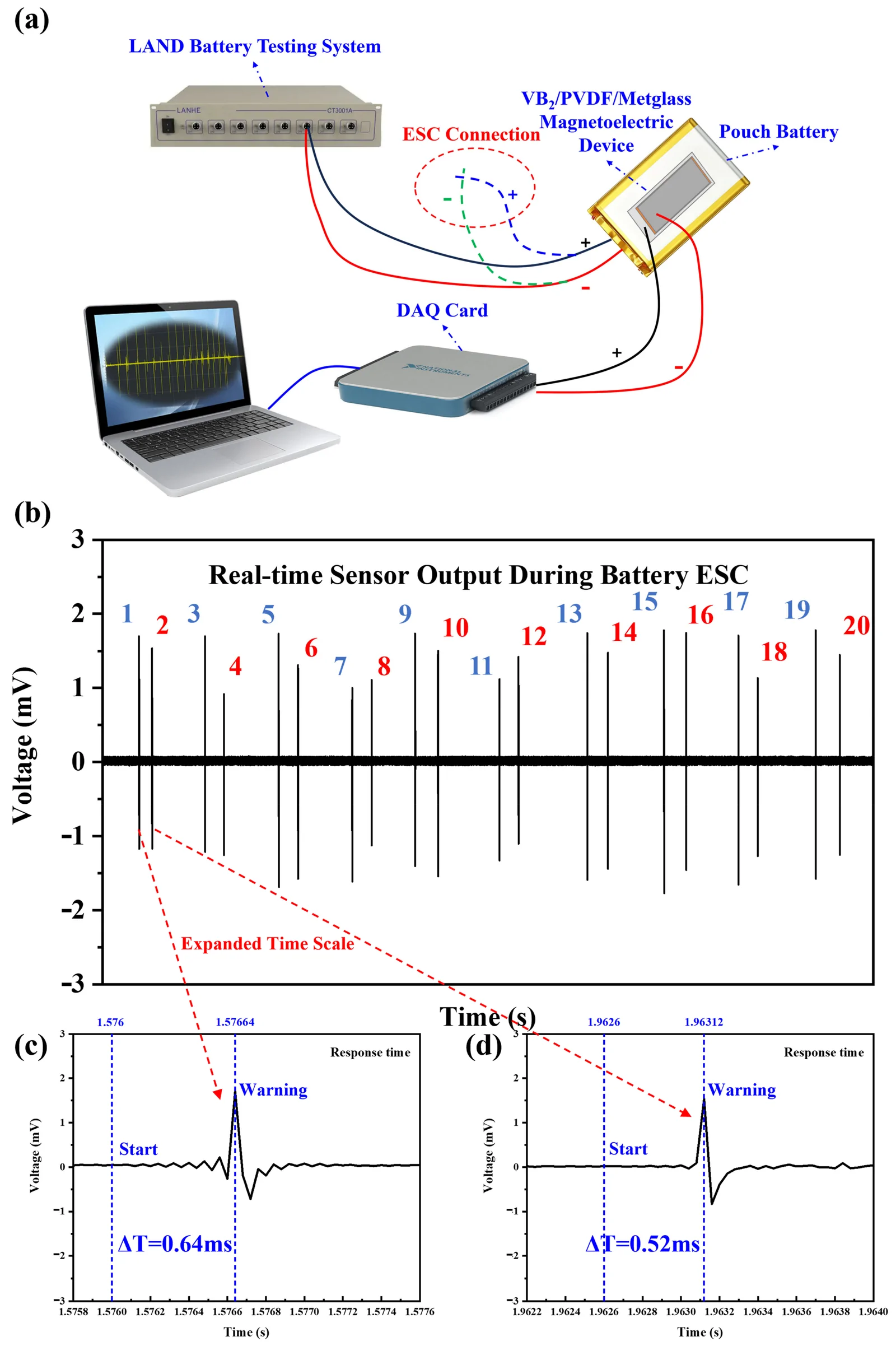
(**a**) Schematic illustration of the VB_2_/PVDF-TrFE/Metglass magnetoelectric device for monitoring external short-circuit (ESC) events in batteries; (**b**) output voltage signals of the device in response to 10 cycles of ESC onset and release; (**c**) enlarged view of the time scale for Signal “1”; (**d**) enlarged view of the time scale for Signal “2”.

**Table 1:** The absorption intensities of VB_2_/PVDF-TrFE at 764.16 cm^−1^ and 839.85 cm^−1^ and the calculated *F_β_* results.

*A*	Absorbance					
	PVDF-TrFE	3 wt% VB_2_/PVDF-TrFE	5 wt% VB_2_/PVDF-TrFE	7 wt% VB_2_/PVDF-TrFE	9 wt% VB_2_/PVDF-TrFE	11 wt% VB_2_/PVDF-TrFE
*A_α_*	0.0650	0.0675	0.0659	0.0598	0.0619	0.0531
*A_β_*	0.3484	0.4251	0.5058	0.5543	0.2916	0.2043
*F_β_*	80.96%	83.34%	85.89%	88.03%	78.91%	75.32%

## Data Availability

The original contributions presented in this study are included in the article. Further inquiries can be directed to the corresponding author.
